# Distribution and prognostic significance of gluconeogenesis and glycolysis in lung cancer

**DOI:** 10.1002/1878-0261.12780

**Published:** 2020-09-01

**Authors:** Elisabeth Smolle, Petra Leko, Elvira Stacher‐Priehse, Luka Brcic, Amin El‐Heliebi, Lilli Hofmann, Franz Quehenberger, Andelko Hrzenjak, Helmut H. Popper, Horst Olschewski, Katharina Leithner

**Affiliations:** ^1^ Division of Pulmonology Department of Internal Medicine Medical University of Graz Austria; ^2^ Institute of Pathology Asklepios Clinic München‐Gauting Germany; ^3^ Diagnostic and Research Institute of Pathology Medical University of Graz Austria; ^4^ Gottfried Schatz Research Center Department of Cell Biology, Histology and Embryology Medical University of Graz Austria; ^5^ Center for Biomarker Research in Medicine (CBmed) Graz Austria; ^6^ Institute of Medical Informatics, Statistics and Documentation Medical University of Graz Austria; ^7^ Ludwig Boltzmann Institute for Lung Vascular Research Graz Austria

**Keywords:** gluconeogenesis, GLUT1, glycolysis, lung cancer, PCK2

## Abstract

Inhibition of glycolysis has been considered as a therapeutic approach in aggressive cancers including lung cancer. Abbreviated gluconeogenesis, mediated by phosphoenolpyruvate carboxykinase (PEPCK), was recently discovered to partially circumvent the need for glycolysis in lung cancer cells. However, the interplay of glycolysis and gluconeogenesis in lung cancer is still poorly understood. Here, we analyzed the expression of GLUT1, the prime glucose transporter, and of PCK1 and PCK2, the cytoplasmic and mitochondrial isoforms of PEPCK, in 450 samples of non‐small cell lung cancer (NSCLC) and in 54 NSCLC metastases using tissue microarrays and whole tumor sections. Spatial distribution was assessed by automated image analysis. Additionally, glycolytic and gluconeogenic gene expression was inferred from The Cancer Genome Atlas (TCGA) datasets. We found that PCK2 was preferentially expressed in the lung adenocarcinoma subtype, while GLUT1 expression was higher in squamous cell carcinoma. GLUT1 and PCK2 were inversely correlated, GLUT1 showing elevated expression in larger tumors while PCK2 was highest in smaller tumors. However, a mixed phenotype showing the presence of both, glycolytic and gluconeogenic cancer cells was frequent. In lung adenocarcinoma, PCK2 expression was associated with significantly improved overall survival, while the opposite was found for GLUT1. The metabolic tumor microenvironment and the 3‐dimensional context play an important role in modulating both pathways, since PCK2 expression preferentially occurred at the tumor margin and hypoxia regulated both, glycolysis and gluconeogenesis, in NSCLC cells *in vitro*, albeit in opposite directions. PCK1/2 expression was enhanced in metastases compared to primary tumors, possibly related to the different environment. The results of this study show that glycolysis and gluconeogenesis are activated in NSCLC in a tumor size and oxygenation modulated manner and differentially correlate with outcome. The frequent co‐activation of gluconeogenesis and glycolysis in NSCLC should be considered in potential future therapeutic strategies targeting cancer cell metabolism.

## Graphical abstract

Gluconeogenesis, mediated by phosphoenolpyruvate carboxykinase (PCK2), promotes anabolic metabolism in lung cancer cells in the absence of glucose. Here we show considerable heterogeneity of the utilization of gluconeogenesis or glycolysis in human non‐small cell lung cancers (NSCLC) and NSCLC metastases and a localization of PCK2 at tumor margins. We identify hypoxia as an important modulator of both pathways in NSCLC cells.

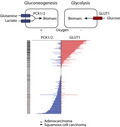

**Abbreviations**
AMPK: AMP‐activated protein kinase
ATF4: AMP‐dependent transcription factor 4
FFPE: formalin‐fixed paraffin‐embedded
GDP: guanosine diphosphate
GLUT1: glucose transporter type 1
GTP: guanosine triphosphate
HE: hematoxylin/eosin
HK: hexokinase
IHC: immunohistochemistry
LDHB: lactate dehydrogenase B
LUAD: lung adenocarcinoma
LUSC: lung squamous cell carcinoma
NSCLC: non‐small cell lung cancer
OAA: oxaloacetate
PCK1: cytosolic isoform of PEPCK
PCK2: mitochondrial isoform of PEPCK
PEP: phosphoenolpyruvate
PEPCK: phosphoenolpyruvate carboxykinase
SLC2A1: solute carrier family 2, facilitated glucose transporter member 1
STK11: serine/threonine‐protein kinase STK11
TCA cycle: tricarboxylic acid cycle
TCGA: The Cancer Genome Atlas
TMA: tissue microarrays

## 1 Introduction

Lung cancer accounts for the majority of cancer‐related deaths worldwide [1]. More than 1.7 million individuals die from lung cancer annually [1]. Non‐small cell lung cancer (NSCLC) represents approximately 85% of all lung carcinoma cases [2]. With novel treatment strategies, the mean overall survival of NSCLC has improved over the last few decades; however, the 5‐year survival is still only 19% for all stages combined [2, 3]. Growing tumors are characterized by *de‐novo* formation of blood vessels; however, this process is highly disorganized resulting in nutrient and oxygen deprivation in certain parts of the tumor [4]. Steep gradients of glucose, oxygen and pH occur within tumors with increasing distance from blood vessels [5]. Accordingly, the extracellular fluid in solid cancers shows glucose levels that are on average half the levels of surrounding normal tissue [6], consistent with a reduced content of glucose in malignant tissues reported in numerous metabolomics studies [7]. Hence, in solid, rapidly growing cancers like NSCLC, cancer cells need to adapt to glucose deprivation. Partially, this occurs by circumventing the glycolytic pathway and instead using some steps of gluconeogenesis from noncarbohydrate precursors to overcome metabolic stress (for review, see [8]). The key enzyme mediating gluconeogenesis, phosphoenolpyruvate carboxykinase (PEPCK), has been recently discovered to replenish glycolytic pools in cancer cells of different origins and thus to mediate abbreviated gluconeogenesis [9, 10, 11, 12, 13]. In numerous studies, PEPCK was shown to enhance tumor cell metabolic flexibility under glucose deprivation [8].

Gluconeogenesis, the formation of glucose from noncarbohydrate precursors like amino acids or lactate, occurs primarily in the liver and kidney [14]. Abbreviated gluconeogenesis, in contrast, results in the biosynthesis of other intermediates, for example, glycerol phosphate [8, 15]. The first step is mediated by PEPCK, which catalyzes the conversion of oxaloacetate (OAA) + GTP to phosphoenolpyruvate (PEP) + GDP + CO_2_. There are two isoforms of PEPCK: the cytoplasmic isoform PCK1, and PCK2, the mitochondrial isoform [16, 17]. In tumor cells of various origins, PCK1 or PCK2 have been found to mediate abbreviated gluconeogenesis, especially under conditions of glucose deprivation, thus contributing to anabolic metabolism [9, 11, 12, 13]. The downstream metabolic pathways fueled by PCK1 or PCK2 in cancer cells include the biosynthesis of the phospholipid glycerol backbone [13], serine [11], or ribose [12, 18]. Thus, PCK1 or PCK2 enables the cells to produce glycolytic intermediates for biosynthetic pathways that are essential for cancer cell proliferation in the absence of glucose. Moreover, PCK2 was shown to directly impact tricarboxylic acid (TCA) cycle function in cancer cells by catabolizing OAA generated from glutamine [19]. Therefore, PEPCK acts as a mediator of cancer cell metabolic adaptation, allowing for glucose‐independent tumor growth and proliferation [8]. Of note, PEPCK inhibition or silencing reduced the growth of tumors in different *in vivo* models [11, 12, 13, 20]. On the other hand, growth inhibitory effects of PEPCK have been found in liver and kidney cancer cells (for review, see [8, 21]).

In NSCLC, PEPCK activity was found to be higher in lung cancer samples compared to normal lung [9]. PCK2 was the prime isoform expressed in NSCLC, while PCK1 levels were found to be generally low [9, 11]. PCK2 expression was upregulated under low glucose conditions in different NSCLC cell lines [9, 11, 13]. Moreover, PCK2 has been shown to be upregulated by cyclic AMP‐dependent transcription factor 4 (ATF4) and endoplasmic reticulum stress [10], and by the Myc superfamily member MondoA [22]. The cues regulating the expression of PEPCK in the complex microenvironment of tumors *in vivo*, however, are still poorly understood.

The 'Warburg' phenotype of cancer cells, characterized by enhanced glycolysis, is mediated mainly by an aberrant expression of glucose transporter type 1 (GLUT1), also known as solute carrier family 2, facilitated glucose transporter member 1 (SLC2A1), a member of the glucose transporter family [23, 24]. Several oncogenic transcription factors have been shown to directly enhance GLUT1 mRNA levels in human cancers [23, 24]. In different cancer types, including NSCLC, GLUT1 overexpression has been linked to a poor prognosis (for review, see [25]). Until today, it is not known how gluconeogenesis and glycolysis interoperate in NSCLC. Furthermore, it remains unknown which subtypes of NSCLC utilize gluconeogenesis. The aim of the present study was to elucidate the complex interaction of the glycolytic and the gluconeogenic pathway in NSCLC.

## 2 Materials and methods

### 2.1 Patients

In the lung adenocarcinoma (LUAD) cohort, 207 male and 135 female patients and in the lung squamous cell carcinoma (LUSC) cohort, 93 male and 15 female patients were included. Samples were all located on tissue microarrays (TMAs). TMAs comprised LUAD as well as LUSC samples. On average three cores were obtained from each individual’s tumor. Tumor samples were obtained from surgical resections performed mainly at the University Hospital of Graz, Austria. Few tumor samples were obtained from surgical procedures performed at a private sanitarium. Clinical and patients’ data are summarized in Table S1. In the metastasis cohort, primary NSCLC of stage IV (metastatic) patients and a total number of 54 NSCLC metastases, partially corresponding, were evaluated. Clinical and patients’ data from the metastasis cohort are summarized in Table S2. Additionally, formalin‐fixed, paraffin‐embedded (FFPE) samples of NSCLC and corresponding normal lung parenchyma from a total number of 29 patients were obtained from the Biobank of the Medical University of Graz. Clinical data was retrieved from the medical records. The study methodologies conformed to the standards set by the Declaration of Helsinki. The study protocol was approved by the ethics review board of the Medical University of Graz.

### 2.2 Immunohistochemistry

Both TMA and whole slide sections were evaluated using immunohistochemistry (IHC). After deparaffinization and antigen retrieval, sections were stained with PCK1 mouse monoclonal antibody (H00005105‐M01, Abnova, Taipei City, Taiwan) diluted 1 : 50; PCK2 rabbit polyclonal antibody (ab137580, Abcam, Cambridge, UK) diluted 1 : 200; lactate dehydrogenase B (LDHB) rabbit monoclonal antibody (EP1565Y, Abcam) diluted 1 : 800, and GLUT1 rabbit polyclonal antibody (ab15309, Abcam) diluted 1 : 100. Isotype antibodies were used as negative controls. Specificity of the PCK2 antibody was validated using stable shRNA‐mediated silencing in cells incorporated into paraffin‐cytoblocks (Fig. S1A). Human liver was used as a positive control tissue for PCK1 staining (Fig. S1B). Slides were analyzed by a lung pathologist (LB), blinded to group assignment and outcome. Staining intensity was defined as negative (0), weak (1), moderate (2), or strong (3). Density of IHC staining was indicated as percentage of positively stained tumor cells (0–100%). A score was calculated as a product of intensity and density, resulting in a range from 0 to 300. The mean score was calculated in the case of multiple cores from a single tumor. Positive IHC staining was defined as a combined score ≥ 1.

### 2.3 Survival analysis

Survival data were provided by the Social Insurance Agency of Austria or were obtained from patients’ records. Survival was calculated from the date of pathological diagnosis until the date of death or last follow‐up. Patients who deceased within the first month after surgery were excluded from the analysis. Overall survival was calculated by means of Kaplan–Meier estimators for PCK1, PCK2, LDHB and GLUT1. As cutoff, an IHC combined score ≥ 1 was used (present or absent staining). Multivariate analysis, including also patients’ tumor stage (6th TNM classification, valid at the time of diagnosis) and tumor grade, age, and gender, was performed using the Cox proportional hazards model. For survival analysis in the TCGA (The Cancer Genome Atlas) LUAD cohort, the median value of expression was used as the cutoff.

### 2.4 CellProfiler analysis

Formalin‐fixed, paraffin‐embedded samples of NSCLC obtained from the Biobank of the Medical University of Graz were stained with the PCK2 antibody. Intratumoral heterogeneity was evaluated using the cellprofiler software, version 3.1.8 [26, 27]. Differences in PCK2 staining between the tumor margin and tumor center were assessed semi‐automatedly. The thickness of the invasive margin of each tumor was arbitrarily set to 1 mm as described previously [28], and the border of the margin was identified by histology. Tumor areas located next to technical (cut) tumor margins were manually excluded from the CellProfiler analysis, since no classification (margin versus center) was possible in these regions. Similarly, areas containing large vessels, bronchi and glandulae bronchiales, or histotechnological artefacts were excluded from the analysis. Macrophages were identified based on their high intensity IHC staining and excluded from the analysis. Using intensity thresholds, areas of PCK2 (IHC) and nuclear (hematoxylin/eosin, HE) staining were identified and total intensity was measured for the invasive margin and the tumor center. The area of PCK2 staining was normalized to the area of nuclei by calculating the ratio of area of PCK2 staining/area of HE staining. For a direct comparison between the margin region and the tumor center, the ratio of area of PCK2 staining/area of HE staining was further normalized to the tumor center. Semi‐automated analysis could be performed in 24 out of 29 slides. Five slides had to be excluded, because of a very small tumor size or because the tumor margin could not be identified.

### 2.5 Cell lines

The human NSCLC cell line NCI‐H23 (H23, ATCC number CRL‐5800) was purchased from American Type Culture Collection (ATCC, Manassas, VA, USA). The human NSCLC cell line A549 (Cat. No. 300114) was purchased from Cell Lines Service (Eppelheim, Germany). NCI‐H460 cells (H460) were a kind gift from Martin P. Barr, Institute of Molecular Medicine, St. James’s Hospital and Trinity College Dublin, Dublin, Ireland, to AH. H23 and H460 cells were cultured in RPMI 1640 supplemented with 10% fetal bovine serum (FBS, Biowest, Nuaillé, France) and antibiotics. A549 cells were cultured in DMEM‐F12 medium (Gibco, Waltham, MA, USA) supplemented with 10% FBS (Biowest) and antibiotics. Mycoplasma tests were performed every three months. Cell line authentication was done for all cell lines by Short Tandem Repeat (STR) analysis using the PowerPlex 16HS System (Promega, Madison, WI).

### 2.6 Low glucose and hypoxia treatment

Cells were plated into 6‐well plates at 150 000 cells per well and let attach for 24 h. After two times washing with phosphate‐buffered saline, cells were incubated for 48 or 96 h in glucose‐ and glutamine free, serum‐free DMEM medium (Gibco) supplemented with different concentrations of glucose and 2 mm glutamine in ambient oxygen or 1% oxygen in the automated Xvivo System G300CL (BioSpherix, Lacona, NY, USA). After 48 h, media were replaced by fresh, pre‐equilibrated media. Exposure to ambient oxygen was prevented throughout the experiment.

### 2.7 Western blot

Cells were lysed on ice in RIPA buffer (Sigma‐Aldrich, St. Louis, MO, USA) containing protease inhibitors. Proteins were separated by sodium dodecyl sulfate‐polyacrylamide gel electrophoresis using the Mini‐PROTEAN^®^ electrophoresis unit (Bio‐Rad, Hercules, CA, USA) and transferred to a PVDF membrane (Bio‐Rad). Immunodetection was performed with a rabbit monoclonal antibody to PCK2 (ab187145, Abcam, diluted 1 : 2000), a mouse monoclonal antibody to β‐actin (Santa Cruz Biotechnology, Santa Cruz, CA, USA diluted 1 : 3000), or a GAPDH antibody linked to peroxidase (Santa Cruz, 1 : 10 000). Peroxidase‐linked secondary antibodies were used at a dilution of 1 : 3000. Peroxidase activity was detected using chemiluminescence detection (SuperSignal West Pico Chemiluminescent Substrate, Thermo Fisher Scientific).

### 2.8 Quantitative real‐time PCR

Total RNA was extracted with the peqGOLD Total RNA Kit (VWR, Vienna, Austria). Total RNA (1 µg) was reverse transcribed in a final volume of 20 µL using the qScript cDNA synthesis kit (Quantabio, Beverly, MA, USA). Real‐time PCR was performed using the LightCycler 480 (Roche, Vienna, Austria) and the QuantiFast SYBR PCR kit (Qiagen, Hilden, Germany). Primers used were as follows: 5’‐CATCCGAAAGCTCCCCAAGTA‐3’ (forward strand) and 5’‐TGGAAATCAGCTGGGGACATC‐3’ (reverse strand) for human *PCK2*; 5’‐AGACGAGAGTTTCCTGGTCTCA‐3’ (forward strand) and 5’‐TTCCGGATCAGAGCCACAAC‐3’ (reverse strand) for *HK2*, 5’‐TGGCATCAACGCTGTCTTCT‐3’ (forward strand) and 5’‐AGCCAATGGTGGCATACACA‐3’ (reverse strand) for GLUT1 (encoded by *SLC2A1*); 5’‐CTACCACATCCAAGGAAGCA‐3’ (forward strand) and 5’‐TTTTTCGTCACTACCTCCCCG‐3’ (reverse strand) for *18S* rRNA. 18S rRNA served as a reference gene, as it showed stable expression. ΔCp was calculated by subtracting the Cp number of the gene of interest from that of 18S rRNA. ΔΔCp values of the control group were subtracted from ΔCp values of the treated group. Gene expression was calculated as 2^ΔΔCp^.

### 2.9 Antibody specificity control by shRNA‐mediated silencing

For stable expression of PCK2 shRNA or nonsilencing shRNA, H23 cells were transfected with different commercially available shRNA plasmids targeting PCK2 or a nonsilencing control shRNA (Qiagen, Hilden, Germany) followed by selection with 1 µg/mL puromycin (Sigma‐Aldrich) as described [13]. Monoclonal subcultures expressing two different PCK2 shRNA constructs (#1 and #2) or control shRNA were generated. For cultivation of cells, puromycin was used at the maintenance concentration of 0.5 µg/ml. During the course of the experiments, puromycin was omitted. Successful silencing was confirmed with western blot and quantitative PCR (qPCR) [13]. Cytoblocks of cells cultivated for 72 h with daily medium replacement in low glucose (0.2 mm) and 10 mm lactate containing serum‐free RPMI medium were generated using human plasma. After clotting by addition of 13.8 mm CaCl_2_ and cell debris, cellblocks were fixed in 4% buffered formaldehyde (pH 7.4), paraffin‐embedded, and stained with PCK2 antibody or isotype antibody. Images are shown in Fig. S1A.

### 2.10 Gene expression analysis in publically available NSCLC datasets

Gene expression data of *PCK1*, *PCK2*, GLUT1 (*SLC2A1*), and *LDHB* were retrieved from different public datasets. TCGA (The Cancer Genome Atlas) gene expression and mutation data for LUAD and LUSC, generated by the TCGA Research Network (https://www.cancer.gov/tcga), was retrieved using the UCSC Xena platform (https://xenabrowser.net/) or via cbioportal (https://www.cbioportal.org/) [29].

### 2.11 Statistical analysis

Data were compiled and analyzed with the software package spss, version 25.0 (Chicago, IL, USA). The standard deviation of expression of the analyzed markers has not been known in different histological subtypes of NSCLC and NSCLC metastases *a priori*. Thus, in this explorative study, sample size calculations were not performed. Group differences were calculated using Mann–Whitney *U* test, two‐sided unpaired Student’s *t*‐test, or one‐group Student’s *t*‐test as appropriate. Spearman’s correlation coefficient was calculated to determine correlations of different markers. *P*‐values smaller than 0.05 were considered significant.

## 3 Results

### 3.1 Gluconeogenesis is more frequent in LUAD than in LUSC and more abundant in small tumors

**Fig. 1:**
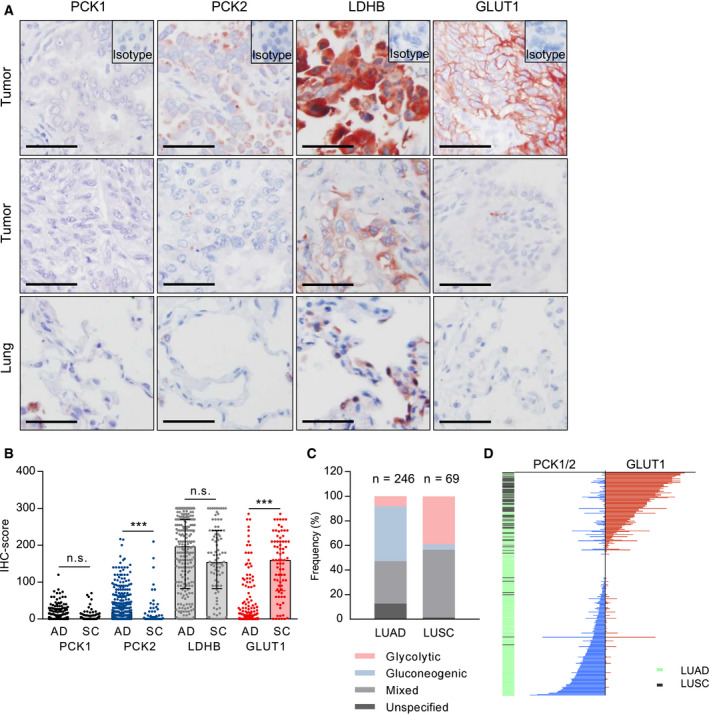
Glycolysis and gluconeogenesis markers in NSCLC and differences according to histology and tumor size. (A) Examples of strong or moderate (1st row) versus weak/negative IHC staining (2nd row) for PCK1, PCK2, LDHB, and GLUT1 in tumors and normal lung tissue (3rd row). Scale bar = 50 µm. (B) Comparison of IHC scores in adenocarcinoma (AD) and squamous cell carcinoma (SC). Group comparisons were performed with the Mann–Whitney U‐test in LUAD and LUSC patients with evaluable PCK1 (*n* = 258/85), PCK2 (*n* = 264/94), LDHB (*n* = 257/92), and GLUT1 staining (*n* = 276/77). *** *P* < 0.001. (C) Frequency of glycolytic (GLUT1 positive, PCK1/2 negative), gluconeogenic (GLUT1 negative, PCK1 or PCK2 positive), and mixed phenotypes (GLUT1 positive and PCK1 or PCK2 positive). Samples without immunopositivity for either PCK1, PCK2, or GLUT1 were defined as 'unspecified'. LUAD, lung adenocarcinoma; LUSC, lung squamous cell carcinoma. (D) Waterfall plot visualizing combined PCK1/2 IHC scores and GLUT1 scores in a total of 317 NSCLC patients.

Our first step was to examine the IHC expression of the two gluconeogenic enzymes (PCK1 and PCK2), and of the glucose transporter mediating glycolysis (GLUT1) in primary NSCLC samples, comparing expression profiles in LUAD versus LUSC. Lactate dehydrogenase B (LDHB), which mediates the interconversion of pyruvate and lactate, and thus contributes to both pathways, was analyzed in parallel. GLUT1 showed a clear membranous localization, while PCK1, PCK2, and LDHB were localized intracellularly (Fig. 1A).

LUAD tumors showed significantly higher expression of PCK2 than LUSC. Conversely, GLUT1 expression was more abundant in LUSC than in LUAD (Fig. 1B). In LUAD, the majority of samples (180; 69%) was positively stained for the gluconeogenic enzyme PCK2, while in the LUSC cohort only 38 (40%) of tumor specimens were PCK2 positive (Table 1). LDHB, which generally showed strong staining in the majority of NSCLC samples, was expressed to a similar extent in LUAD and in LUSC. PCK1 staining tended to be weak (Fig. 1A and B, Table 1). Interestingly, PCK2 and GLUT1 expression showed a clear negative correlation (*r *= −0.2, *P* = 0.001) in LUAD, but not in LUSC. This indicates that in highly glycolytic tumors, PCK2 expression tends to be low, and *vice versa*. Of note, PCK1 and PCK2 were significantly correlated in LUSC (*r* = 0.22, *P* = 0.043), but not in LUAD.

**Table 1:** IHC staining results in primary NSCLC on tissue microarrays

	LUAD	LUSC
PCK1	*n* = 258	*n* = 85
positive	109 (42%)	43 (51%)
negative	149 (58%)	42 (49%)
PCK2	n = 264	n = 94
positive	180 (69%)	38 (40%)
negative	84 (31%)	56 (60%)
LDHB	n = 257	n = 92
positive	233 (91%)	84 (91%)
negative	24 (9%)	8 (9%)
GLUT1	n = 276	n = 77
positive	113 (41%)	72 (94%)
negative	163 (59%)	5 (6%)

LUAD, lung adenocarcinoma; LUSC, lung squamous cell carcinoma.

Importantly, in 34.6% of LUAD and in 55.1% of LUSC samples a 'mixed' phenotype with expression of GLUT1 as well as PEPCK (PCK1 or PCK2) was present (Fig. 1C). Purely glycolytic tumors (i.e., GLUT1‐positive tumors lacking PCK1 or PCK2) were found in 8.1% and 39.1% in LUAD and LUSC, respectively. A gluconeogenic phenotype with positive PCK1 or PCK2 expression but no evidence of GLUT1 expression was observed in 44.7% (LUAD) and in 4.3% (LUSC) of cases (Fig. 1C). In some samples of LUAD and LUSC, neither GLUT1 nor PCK1 or PCK2 expression was present ('unspecified' in Fig. 1C). In these tumors, expression of the enzymes was probably below detection limits. We also cannot exclude the contribution of GLUT3, a GLUT isoform that has been shown to be expressed in NSCLC albeit at lower levels than GLUT1 [30]. The waterfall plot in Fig. 1D with each line representing one patient visualizes the inverse correlation of GLUT1 and PCK1/2, the preference for glycolysis in LUSC, and the occurrence of co‐expression of glycolysis and gluconeogenesis markers in NSCLC.

**Fig. 2:**
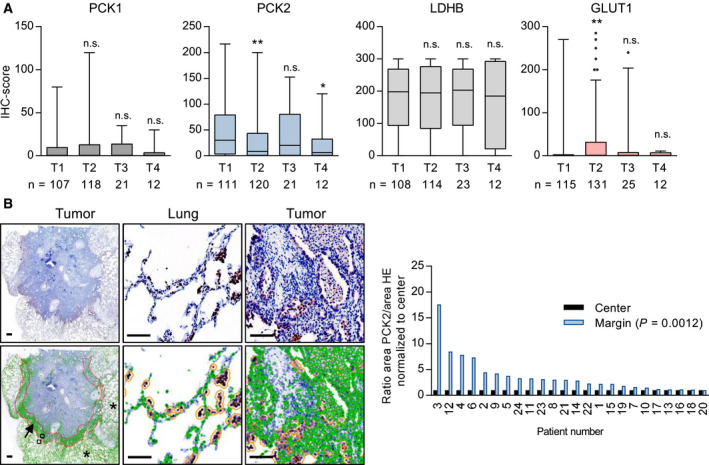
Intratumoral heterogeneity in gluconeogenesis gene expression and relation to tumor size. (A) Differences in glycolysis and gluconeogenesis gene expression according to T stage *P < 0.05, **P < 0.01 on Mann–Whitney *U*‐test versus T1. n.s., not significant; (B) Expression intensity of PCK2 as compared between tumor margin (arrow) and center. Normal lung tissue is indicated with asterisks. Scale bar = 100 µm. Strongly stained macrophages (orange circles) were automatically excluded by setting an appropriate cutoff value. Right: Results of automated analysis by means of the cellprofiler software.

When we analyzed the correlation of glycolytic or gluconeogenic enzyme expression with tumor‐related parameters, we found that in LUAD (*r* = −0.2, *P* = 0.0014) and in LUSC (*r* = 0.13, *P* = 0.03), PCK2 expression significantly decreased with increasing tumor size. By contrast, GLUT1 showed a positive correlation with tumor size and tumor grade (*r* = 0.38, *P* < 0.001 (Fig. 2A and Fig. S2). Female gender was significantly associated with higher PCK2 IHC scores within the LUAD subtype (median score 25, compared to a median score of 10 in males, *P* = 0.02 on Mann–Whitney *U*‐test), while the opposite relation was found for GLUT1 (*P* = 0.02). Age at diagnosis showed a weak negative correlation with GLUT1 (*r *= −0.14, *P* = 0.02) and a weak positive correlation with PCK2 (*r* = 0.13, *P* = 0.04) in LUAD.

### 3.2 PCK2 expression preferentially occurs at tumor margins

In order to clarify whether gluconeogenic and glycolytic cancer cells are found in different regions of the tumor, we analyzed complete sections of 24 FFPE NSCLC samples. Differences in PCK2 in the invasive tumor margin versus the tumor center were examined using automated analysis. As intratumoral and alveolar macrophages were strongly stained, they were excluded from the analysis. In line with observations made by visual inspection, a clearly stronger PCK2 staining was found in the tumor margin, as opposed to the tumor center (*P* = 0.0012; Fig. 2B). GLUT1 or LDHB expression showed no differences between the tumor center and periphery, according to the analyzing pathologist. In this small cohort, PCK1 expression was low, and a possible differential expression in the center vs. margin could not be determined.

### 3.3 High expression of PCK2 and low expression of GLUT1 are associated with a favorable outcome in LUAD

**Fig. 3:**
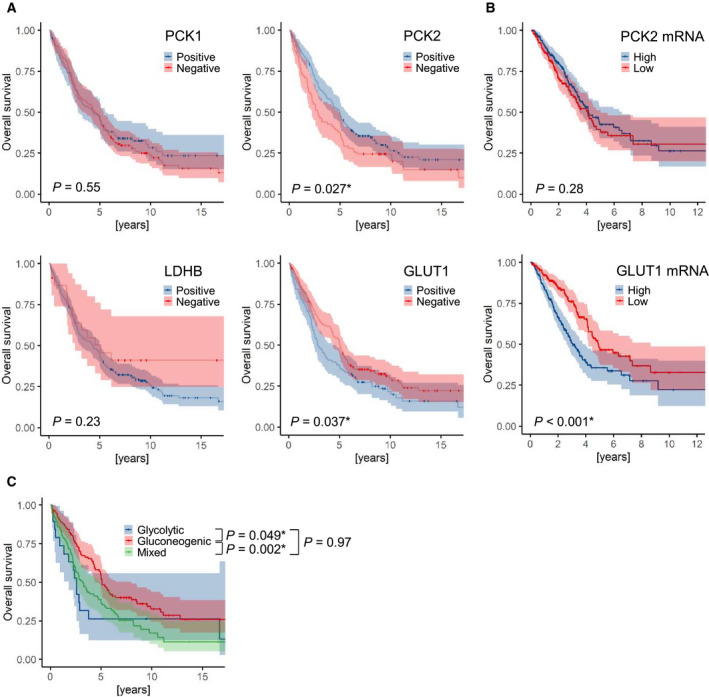
Association of gluconeogenic enzyme expression in lung adenocarcinoma with overall survival. (A) Kaplan–Meier survival functions +/‐ 95% confidence interval in LUAD patients with positive or negative PCK1, PCK2, LDHB and GLUT1 staining (cutoff = 1). (B) Kaplan–Meier estimators of the publicly available TCGA LUAD data for *PCK2* and GLUT1 (*SLC2A1*) mRNA, respectively (*n* = 491; data were retrieved via the UCSC Xena platform, https://xenabrowser.net/). Median values were used as the cutoff. (C) Overall survival in LUAD patients according to the metabolic phenotype. (A–C) Differences in survival were calculated using the Logrank test.

When we assessed the correlation of glycolysis and gluconeogenesis markers in the large LUAD and LUSC cohorts, we found that overall survival was significantly longer in LUAD patients whose tumors featured detectable PCK2 staining (Fig. 3A). Conversely, in LUAD patients whose tumors were positive for GLUT1, overall survival was significantly worse (Fig. 3A). We found a similar significant association of *GLUT1* expression with poor outcome in the TCGA LUAD dataset (*n* = 491); however, in the case of *PCK2* no significant association was found (Fig. 3B). Accordingly, in LUAD glycolytic and mixed tumors were associated with a significantly worse overall survival than gluconeogenic tumors (Fig. 3C). No significant difference was found when comparing glycolytic and mixed LUAD (Fig. 3C). In LUSC, none of the markers or phenotypes were significantly associated with survival (data not shown); however, the sample size was smaller in this cohort (Table S1). *PCK2* expression was significantly associated with a favorable outcome in LUAD also in multivariate survival analysis including tumor stage, grade, age at diagnosis, and gender (Table S3).

In normal lung, a weak‐to‐moderate staining for PCK2 was found in bronchial epithelial cells, but not in type I alveolar epithelial cells. This heterogenous pattern precluded a quantitative assessment of PCK2 immunopositivity in tumor tissue versus normal lung. However, when we assessed mRNA abundance in the TCGA LUAD and LUSC datasets, we found a downregulation of *PCK2* and an upregulation of *LDHB* and GLUT1 (*SLC2A1*) in the cancerous tissue as opposed to normal lung tissue (Fig. S3AB). In LUSC, also a lower expression of *PCK1* in tumors compared to normal lungs was observed (Fig. S3B).

### 3.4 Gluconeogenesis is enhanced in NSCLC metastases as compared to primary tumors

**Fig. 4:**
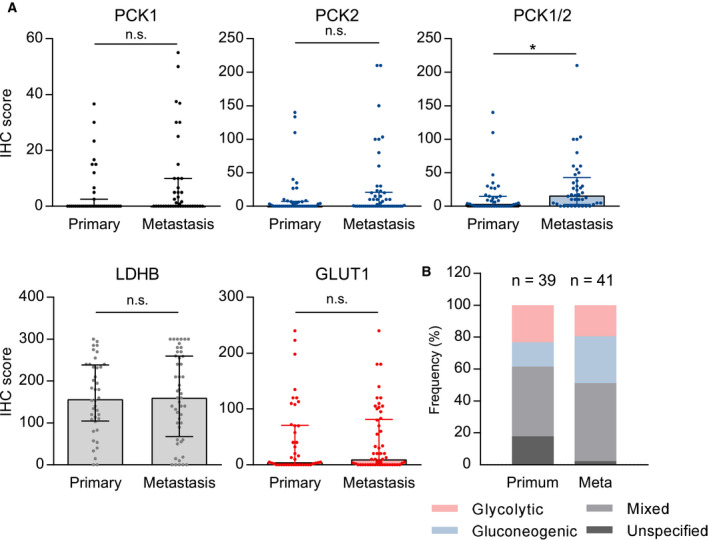
Gluconeogenesis and glycolysis enzyme expression in primary tumors and metastases. (A) A total number of 42 primary tumors from metastatic patients and 54 samples of NSCLC metastases were analyzed. No significant differences in IHC expression between primaries and metastases were observed in the separate analysis of PCK1, PCK2, LDHB, and GLUT1. The combined score of PCK1 and PCK2 (sum of the two scores) is significantly higher in the metastases. Group comparisons were performed with the Mann–Whitney *U*‐test in primary tumors and metastases with evaluable PCK1 (*n* = 39/41), PCK2 (*n* = 41/50), LDHB (*n* = 40/48), GLUT1 staining (*n* = 42/53), or PCK1/2 combined scores (*n* = 39/41). *P < 0.05. (B) distribution of glycolytic, gluconeogenic, and mixed phenotypes. Samples without immunopositivity for either PCK1, PCK2, or GLUT1 were defined as 'unspecified'.

Next, we wanted to explore, whether differences in glycolytic and gluconeogenic enzyme expression profiles between NSCLC primary tumors and metastases exist. The metastases (*n* = 54), which were derived mainly from brain metastases, were present on TMAs along with primary tumors from metastatic NSCLC patients (*n* = 42) with similar histopathological characteristics (Table S2). No significant differences in IHC expression between primaries and metastases were observed in the separate analysis of PCK1, PCK2, LDHB, and GLUT1. However, the combined score of PCK1 and PCK2 was significantly higher in the metastases (*P* = 0.01, Fig. 4A). When the different phenotypes where determined on the basis of PCK1, PCK2, and GLUT1 expression, an increase in the gluconeogenic and mixed phenotypes was found in the metastases (Fig. 4B).

### 3.5 PCK2 expression is decreased in KRAS‐mutated LUAD, and GLUT1 is over‐expressed in TP53‐mutated tumors

In the large LUAD TCGA cohort, *KRAS* mutant tumors displayed a slightly but significantly reduced *PCK2* expression, while *GLUT1* expression was reduced in LKB1 (*STK11*) mutant tumors and enhanced in *TP53* mutant tumors (Fig. S4). The overall effect of cancer driver mutations on *GLUT1* and *PCK2* expression appeared limited and may only partially explain the observed interpatient variability.

### 3.6 Hypoxia differentially impacts gluconeogenesis and glycolysis

**Fig. 5:**
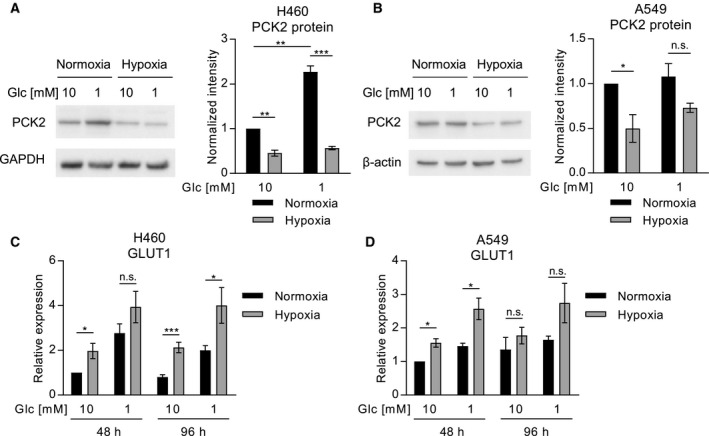
PCK2 and GLUT1 expression are modulated by hypoxia in lung cancer cell lines. Expression of PCK2 protein in H460 (A) and A549 (B) lung cancer cell lines, cultured in high glucose (10 mm) or low glucose (1 mm) media, in normoxia or hypoxia (1% oxygen). Beta‐actin and GAPDH were used as loading controls. Integrated density was normalized to first sample and the loading control. (C and D) GLUT1 (*SLC2A1*) expression analyzed by quantitative PCR in H460 (C) and A549 (D) cells. Results, displayed as mean +/‐ SEM, were obtained in four (A, B), six (C), or five (D) independent experiments. Group comparisons were performed using Student’s *t*‐test or one‐group Student’s *t*‐test as applicable. *P < 0.05, **P < 0.01, ***P < 0.001.

It is well known that besides cell‐intrinsic factors, like oncogenic driver mutations or tumor histology, the metabolic microenvironment and the availability of nutrients and oxygen are critical regulators of cancer cell metabolism [31]. Glycolysis is enhanced by hypoxia, partly via the upregulation of glycolytic genes [31, 32]. It has previously been described that hypoxia plays a role in NSCLC [33, 34]. In breast cancer cells, hypoxia reduced the expression of PCK2 [35], suggesting a possible interaction of gluconeogenesis and hypoxia. Therefore, we investigated the impact of hypoxia on PCK2 and GLUT1 in NSCLC cell lines and their modulation by different glucose levels. To this end, we cultured two lung cancer cell lines, H460 and A549, in high glucose (10 mm) or low glucose (1 mm) media under normoxia or hypoxia, respectively, to assess PCK2 and GLUT1 expression. PCK2 showed a trend for increased expression levels upon mild glucose starvation compared to high glucose medium. Hypoxia led to a significant downregulation of PCK2 in H460 cells compared to normoxic conditions, both in high and low glucose media (Fig. 5A). Similar findings were obtained in A549 cells (Fig. 5B). *PCK1* mRNA was detected only at low levels in the two cell lines. Interestingly, *PCK2* mRNA was only insignificantly regulated by hypoxia (Fig. S5), suggesting a possible post‐translational mechanism, which has been described for PCK1 [36], but has not yet been investigated for the regulation of PCK2. Conversely, mRNA levels of *GLUT1* (Fig. 5C,D) as well as hexokinase 2 (*HK2,* Fig. S5), known hypoxia‐regulated glycolytic genes, were significantly elevated under hypoxic conditions.

## 4 Discussion

With this study, we aimed to shed light on the intra‐ and intertumoral heterogeneity of glycolytic and gluconeogenic gene expression in primary NSCLC and NSCLC metastases. We found that gluconeogenesis and glycolysis are inversely correlated in NSCLC. PCK2 decreased with increasing tumor size, especially in the LUAD subtype. When we analyzed the abundance of gluconeogenesis and/or glycolysis markers in sections of complete tumors we found an unexpected upregulation of PCK2 at the tumor margins, compared to tumor centers, while for GLUT1 no preference was found. We show that PCK2 expression was decreased by hypoxia in lung cancer cells *in vitro*, while the opposite was true for glycolysis marker GLUT1. Since the tumor center has been shown to be more hypoxic than the tumor margin [4], central hypoxia might contribute to the decrease in PCK2 expression in this compartment. The independence of GLUT1 expression from the localization in the tumor, however, might be related to the fact that oncogenes are important drivers of GLUT1 expression in addition to microenvironmental factors [23, 24]. Our results suggest that tumors may switch from a more gluconeogenic to a more glycolytic phenotype with increasing tumor size. Hypoxia, which is known to increase with tumor size, might contribute to this adaptive process.

Metabolic heterogeneity is increasingly being identified in malignant tumors, partially related to the tissue of origin and to tumor histology [37]. In our study, PCK2 expression was found to be significantly higher in LUAD samples, as compared to LUSC. Conversely, for GLUT1, higher average IHC scores were found in LUSC samples. The latter finding is in line with previously published data [38, 39, 40, 41]. An enhanced glycolytic activity of LUSC compared to LUAD has recently been shown in patients using stable isotope‐resolved metabolomics [42]. Furthermore, LUSC cells displayed a highly glucose‐dependent metabolic phenotype, whereas LUAD cell lines were less glucose‐dependent and insensitive to silencing of GLUT1 [41]. *In vivo* glucose uptake as measured by ^18^F‐fluorodeoxyglucose‐positron emission tomography was found to be higher in LUSC than in LUAD [40, 41]. Conversely, tumor vascularity measured by dynamic contrast enhanced magnetic resonance imaging [43] and vascular density [40] have been shown to be higher in LUAD than in LUSC. Thus, the presence of hypoxia could not only be responsible for the lower expression of PCK2 in the tumor center and in larger tumors, but also for the observed histology‐related differences. The role of oxygen as a regulator of the metabolic adaptation from gluconeogenesis to glycolysis in growing tumors, however, is yet to be clarified and should be addressed in studies involving 3D models or experimental tumors *in vivo*.

Our survival data show that a positive PCK2 expression is a favorable prognostic parameter in LUAD, while a positive GLUT1 expression is associated with a poor prognosis. The prognostic significance of GLUT1 in solid cancers including NSCLC is well established. In a meta‐analysis comprising more than twenty studies, GLUT1 overexpression was shown to be significantly associated with an unfavorable overall survival in patients with NSCLC [44]. Only limited data exist on the association of PCK2 expression and prognosis in different cancer entities. High PCK2 expression predicted a worse overall survival in prostate cancer [19]. Here, we show that the mixed phenotype, exhibiting GLUT1 as well as PCK1/2 expression was significantly associated with a worse overall survival in LUAD compared to the purely gluconeogenic phenotype, while no significant difference was found between the mixed and the purely glycolytic phenotypes (Fig. 3C). Thus, flexible activation of both glycolysis and gluconeogenesis in lung cancer cells might contribute to cancer progression and might enhance aggressiveness compared to activation of gluconeogenesis alone.

The combined PCK1 and PCK2 score was significantly elevated in NSCLC metastases compared to primary cancers, while the glycolysis marker GLUT1 showed no differential expression in metastases versus primary tumors. As a limitation, primary samples from metastatic patients and metastases were derived from different sets of patients and only a subset was corresponding. The low rate of surgeries of NSCLC metastases or biopsies taken from concurrent primaries and metastases precluded the immunohistochemical analysis of a larger number of corresponding samples. In previous studies, an elevation of PCK1 or PCK2 was found in metastases of solid tumors compared to primary tumors (for review, see [8]). Prostate cancer from metastatic patients showed higher PCK2 expression levels than prostate cancer tissue from nonmetastatic patients [19]. Accordingly, tumor‐initiating cells (TICs) from prostate cancer showed higher PCK2 levels than the parental cells and PCK2 silencing reduced TIC numbers by affecting levels of reactive oxygen species and protein acetylation [19]. The metabolic microenvironment has been shown to differ between metastases and primary tumors and between distinct metastatic sites (for review, see [45]). Whether PCK1 and PCK2 play a mechanistic role in cancer cell adaptation to the metabolic microenvironment in NSCLC metastases and whether the size of the metastases, their oxygenation status or other factors in the metastatic microenvironment influence PCK1/2 abundance, however, remains to be elucidated in future studies.

The lack of PCK1 or PCK2 expression in some NSCLCs leads to the question, how lung cancer cells remain viable and maintain anabolic biosynthesis in these tumors, if blood perfusion drops. Autophagy, a self‐digestion mechanism tightly regulated by nutrient levels via AMP‐activated protein kinase (AMPK), has been shown to be important for adaptation of cancer cells to starvation [46]. Besides autophagy, also micropinocytosis, the scavenging and utilization of extracellular material, may provide fuels and building blocks for cell growth in glucose‐limited environments (for review, see [47]). This mechanism is known to be activated by mutant KRAS in lung tumors [47]. Interestingly, KRAS mutant LUAD showed reduced levels of PCK2 in our study. The interaction of micropinocytosis or autophagy and gluconeogenesis in starved lung cancer cells remains yet to be studied.

Most cancer cells display enhanced glucose uptake and utilization of glycolytic intermediates for anabolic biosynthetic processes and regeneration of reducing equivalents for growth and antioxidant defense. Still, attempts to target glycolysis as a therapeutic tool in cancer have shown limited clinical success [48]. In our study, we found that a large proportion of NSCLCs exhibits a mixed glycolytic and gluconeogenic phenotype. Gluconeogenesis provides glycolytic intermediates in cancer cells, if glucose availability is limited or if proximal glycolysis is inhibited. Our study shows that gluconeogenesis is frequently activated along with glycolysis in NSCLC, and thus, activation of gluconeogenesis and utilization of noncarbohydrate precursors for biosynthetic reactions may partially explain a lack of efficacy of glycolysis inhibitors. Of note, a recently developed PEPCK inhibitor efficiently reduced the growth of different xenografts with no apparent toxicity in mice [20]. Inhibition of glycolysis and gluconeogenesis simultaneously would potentially allow to target different tumor niches and compartments.

## 5 Conclusions

As a conclusion, this study reveals a complex metabolic landscape in NSCLC with heterogenous activation of glycolysis or gluconeogenesis, considerable histology‐related differences and interpatient variability. Our findings show that glycolysis and gluconeogenesis act in concert in NSCLC. Whether targeting both pathways could be a potential anticancer strategy remains to be addressed in future studies.

## Conflict of interest

The authors have no conflicts of interest to declare.

## Authors contributions

KL, ES, and HO designed the study. KL, PL, AH, and AE developed methods. ES, ES‐P, HHP, LB, KL, AE, and LH contributed to data acquisition. FQ, KL, and ES analyzed data. ES, KL, PL, ES‐P, LB, LH, FQ, AH, HHP, and HO drafted or revised the manuscript. KL and HO supervised the study.

## Supporting information


**Fig S1.** Positive and negative controls for PCK1 and PCK2 immunohistochemistry.
**Fig S2.** IHC scores of gluconeogenesis and glycolysis enzymes in relation to T‐stage in LUSC.
**Fig S3.** Expression of gluconeogenesis and glycolysis enzymes in tumor tissue and non‐involved lung tissue from NSCLC TCGA datasets.
**Fig S4.** PCK2 and GLUT1 mRNA expression in LUAD featuring mutations of commonly mutated genes versus wild‐type samples.
**Fig S5.** PCK2 and HK2 mRNA abundance in NSCLC cell lines under hypoxia and normoxia.

**Table S1.** Patients’ characteristics.
**Table S2.** Patients’ characteristics (samples of primary NSCLC and NSCLC metastases located on tissue microarrays).
**Table S3.** Multivariate survival analysis in LUAD.
