# Supplementary figures and images for: Distribution and prognostic significance of gluconeogenesis and glycolysis in lung cancer
Source: Mol Oncol. 2020 Sep 1;14(11):2853–67. doi: 10.1002/1878-0261.12780 (PMC7607181; doi:10.1002/1878-0261.12780)

Suppl. Figure 1

A

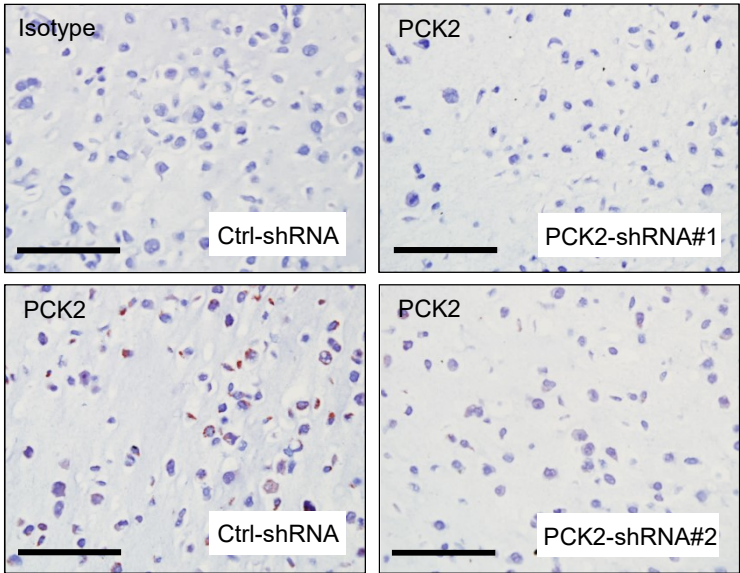

B

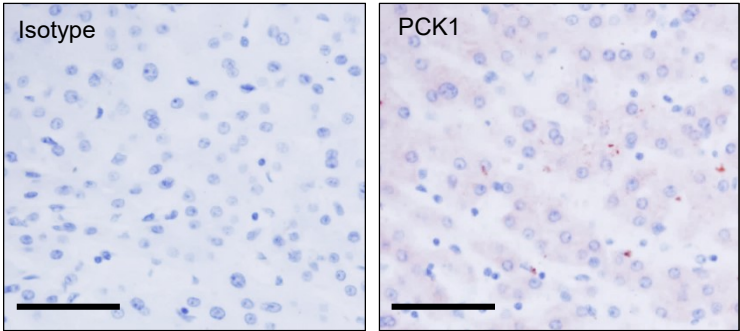

Suppl. Figure 2

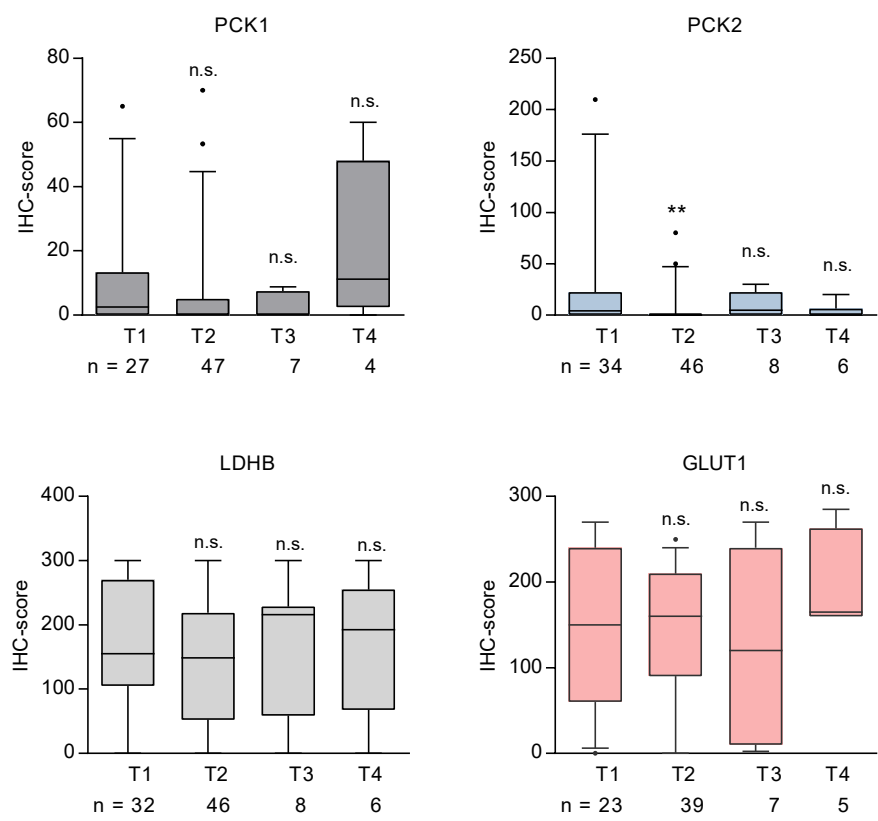

Suppl. Figure 3

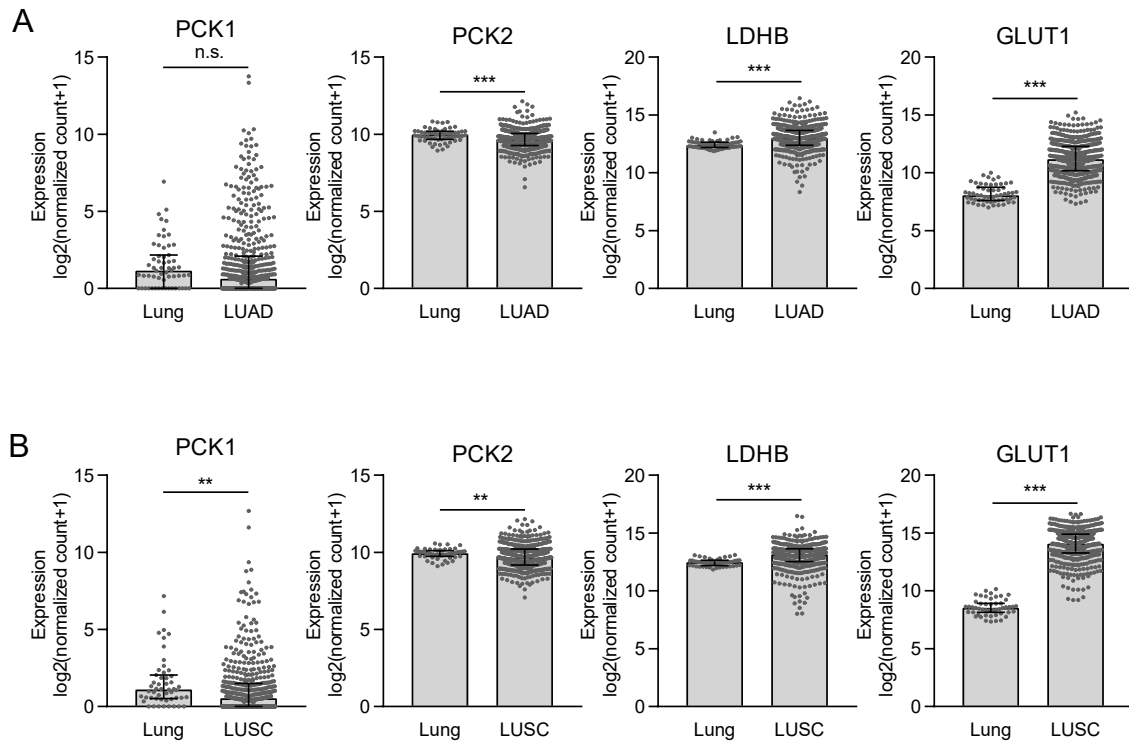

Suppl. Figure 4

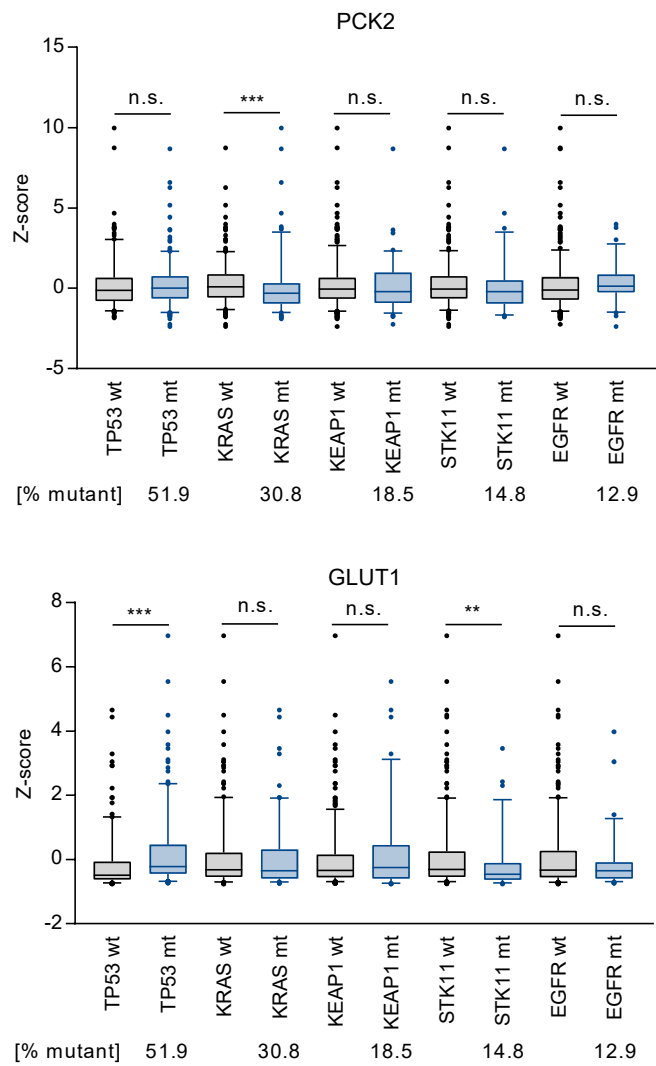

Suppl. Figure 5

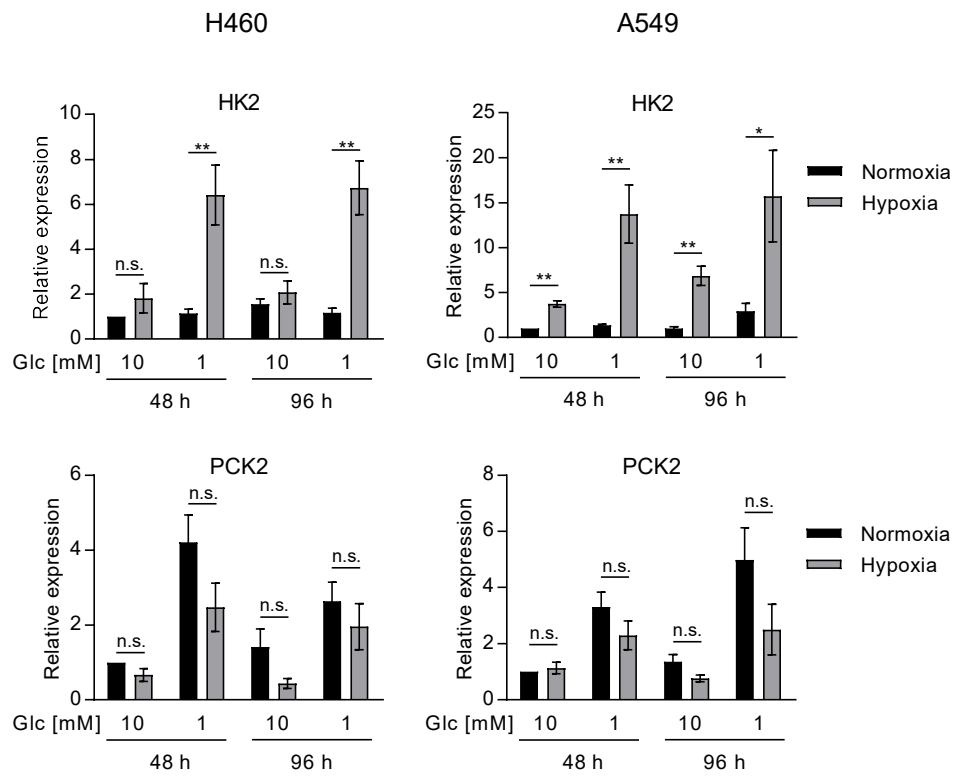

Supplement: Supplementary file 1 — Fig S1. Positive and negative controls for PCK1 and PCK2 immunohistochemistry. Fig S2. IHC scores of gluconeogenesis and glycolysis enzymes in relation to T‐stage in LUSC. Fig S3. Expression of gluconeogenesis and glycolysis enzymes in tumor tissue and non‐involved lung tissue from NSCLC TCGA datasets. Fig S4. PCK2 and GLUT1 mRNA expression in LUAD featuring mutations of commonly mutated genes versus wild‐type samples. Fig S5. PCK2 and HK2 mRNA abundance in NSCLC cell lines under hypoxia and normoxia. [file MOL2-14-2853-s001.pdf]
