# Supplementary material for: Distribution and prognostic significance of gluconeogenesis and glycolysis in lung cancer
Source: Mol Oncol. 2020 Sep 1;14(11):2853–67. doi: 10.1002/1878-0261.12780 (PMC7607181; doi:10.1002/1878-0261.12780)
Supplement: Supplementary file 2 — Table S1. Patients’ characteristics. Table S2. Patients’ characteristics (samples of primary NSCLC and NSCLC metastases located on tissue microarrays). Table S3. Multivariate survival analysis in LUAD. [file MOL2-14-2853-s002.pdf]

Supplementary Table S1. Patients' characteristics

CHARACTERISTICS	ADENOCARCINOMA	N (%)	SQUAMOUS CELL CARCINOMA	N (%)
**All Cases**		342		108
**Gender**				
	Male	207 (60.5)	Male	93 (86)
	Female	135 (39.5)	Female	15 (13.9)
**Age (yr)**				
	Median	64	Median	64
	Range	16-86	Range	37-84
**Stage (TNM 6th)**				
	IA	105 (30.7)	IA	33 (30.6)
	IB	74 (21.6)	IB	24 (22.2)
	IIA	35 (10.2)	IIA	8 (7.4)
	IIB	69 (20.2)	IIB	29 (26.9)
	IIIA	33 (9.6)	IIIA	6 (5.6)
	IIIB	15 (4.4)	IIIB	8 (7.4)
	IV	11 (3.2)	IV	0 (0)

Supplementary Table S2. Patients' characteristics (samples of primary NSCLC and NSCLC metastases located on tissue microarrays)

CHARACTERISTICS	PRIMARY LUNG TUMORS	N (%)
**All Cases**		42
**Gender**		
	Male	27 (64)
	Female	15 (36)
**Age (yr)**		
	Median	59
	Range	37-79
**Histopathology**		
	Adenocarcinoma	25 (59)
	Squamous cell carcinoma	4 (10)
	Large cell	9 (21)
	Mixed / Other	4 (10)
**Tumor grade**		
	1	5 (12)
	2	14 (33)
	3	20 (48)
	4	1 (2)
	unspecified	2 (5)
**Tumor stage**	IV	42 (100)

CHARACTERISTICS	METASTASES	N (%)
**All Cases**		54
**Gender**		
	Male	35 (65)
	Female	19 (35)
**Age (yr)**		
	Median	60
	Range	36-79
**Histopathology (of primary tumor)**		
	Adenocarcinoma	35 (65)
	Squamous cell carcinoma	9 (17)
	Large cell	6 (11)
	Mixed / Other	4 (7)
**Tumor grade (of primary)**		
	1	5 (9)
	2	20 (37)
	3	25 (46)
	4	1 (2)
	unspecified	3 (6)
**Type of metastasis**		
	Brain	48 (89)
	Lymph node	1 (2)
	Other	5 (9)

Supplementary Table S3. Multivariate survival analysis in LUAD

**IHC SCORE**	**RELATIVE RISK**	**95% CI**	***P***
G	1.27	1.07-1.52	0.008*
T	1.25	1.05-1.49	0.011*
N	1.47	1.22-1.77	< 0.001*
M	1.4	0.67-2.91	0.37
Gender (f. v.s. m.)	0.58	0.44-0.77	< 0.001*
Age	1.02	1.002-1.03	0.021*
PCK1 (score)	1.002	0.99-1.01	0.58
PCK2 (score)	0.995	0.992-0.999	0.006*
LDHB (score)	1.001	0.9992-1.002	0.42
GLUT1 (score)	0.9996	0.997-1.003	0.78

G, tumor grade; *significant at $P<0.05$
